# Antibacterial Action of Nanoparticle Loaded Nanocomposites Based on Graphene and Its Derivatives: A Mini-Review

**DOI:** 10.3390/ijms21103563

**Published:** 2020-05-18

**Authors:** Ana María Díez-Pascual

**Affiliations:** Department of Analytical Chemistry, Physical Chemistry and Chemical Engineering, Faculty of Sciences, Institute of Chemistry Research “Andrés M. del Río” (IQAR), University of Alcalá, Ctra. Madrid-Barcelona, Km. 33.6, 28871 Alcalá de Henares, Madrid, Spain

**Keywords:** antibacterial activity, nanocomposites, bacterial infection, antibiotic resistance, graphene, graphene oxide, reduced graphene oxide

## Abstract

Bacterial infections constitute a severe problem in various areas of everyday life, causing pain and death, and adding enormous costs to healthcare worldwide. Besides, they cause important concerns in other industries, such as cloth, food packaging, and biomedicine, among others. Despite the intensive efforts of academics and researchers, there is lack of a general solutions to restrict bacterial growth. Among the various approaches, the use of antibacterial nanomaterials is a very promising way to fight the microorganisms due to their high specific surface area and intrinsic or chemically incorporated antibacterial action. Graphene, a 2D carbon-based ultra-thin biocompatible nanomaterial with excellent mechanical, thermal, and electrical properties, and its derivatives, graphene oxide (GO) and reduced graphene oxide (rGO), are highly suitable candidates for restricting microbial infections. However, the mechanisms of antimicrobial action, their cytotoxicity, and other issues remain unclear. This mini-review provides select examples on the leading advances in the development of antimicrobial nanocomposites incorporating inorganic nanoparticles and graphene or its derivatives, with the aim of providing a better understanding of the antibacterial properties of graphene-based nanomaterials.

## 1. Introduction

Bacterial growth is an increasing issue in everyday life, highly responsible for noteworthy harm in several industries, including cloth, water treatment, biomedicine, and food packaging [1]. Concerns related to the appearance of microorganisms that have become resistant to antibiotics have recently encouraged the search for new treatments of infectious diseases. For instance, some Gram-positive (e.g., *Staphylococcus aureus*) and Gram-negative (*Escherichia coli, Pseudomonas aeruginosa*) bacteria have turned into multi-drug resistant (MDR) pathogens and are one of the leading causes of hospital-acquired infections. Therefore, new, cheap and effective antimicrobial agents for controlling bacterial activity are urgently needed, and nanomaterials constitute a very promising approach.

Many nanomaterials present antimicrobial properties that are not found in their micro/macro-counterparts, like occurs with silver nanoparticles (Ag NPs), which are currently present in wound dressings, coatings for medical devices, and even in food packages. However, commercial applications of Ag NPs are frequently hindered by their agglomeration and consequent loss of antibacterial activity [2].

**Figure 1:**
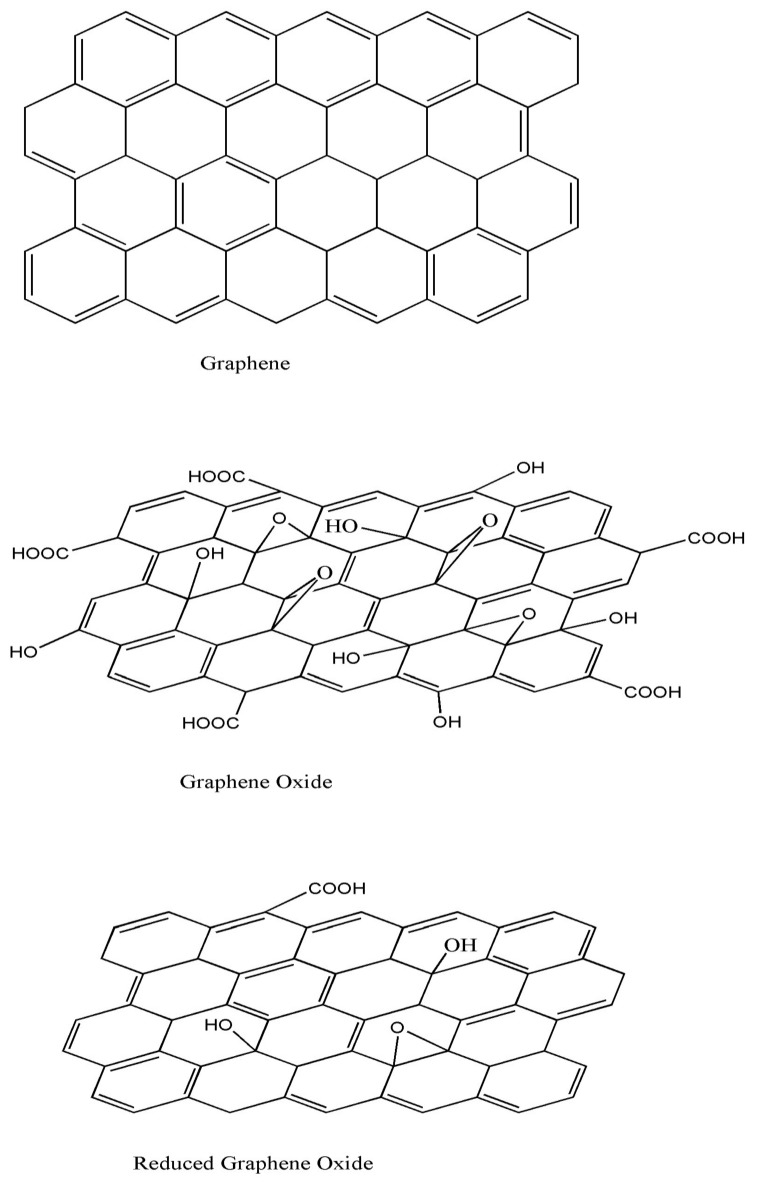
Schematic illustration of the chemical structure of graphene (G) and its derivatives, graphene oxide (GO) and reduced graphene oxide (rGO).

Carbon can be found in the form of different allotropes, with different shapes and properties. Amongst them, graphene (G), a single monolayer of graphite, and its derivatives graphene oxide (GO) and reduced graphene oxide (rGO) are important candidates to be used as antibacterial agents. G consists of a flat, atomically thick single layer of sp^2^ carbon atoms forming a honeycomb structure (Figure 1). It displays exceptional thermal, mechanical, and electronic properties [3]; very high electron mobility (15,000 cm^2^/V × s) and very large surface area (2630 m^2^/g) [4]; an in-plane thermal conductivity close to 5000 W/m × K [3]; and electrical conductivity of 9.6 × 10^7^ S/m. Moreover, G is one of the strongest materials ever known, with Young modulus of around 1 TPa [5], ultimate strength of 130 GPa, and breaking strength of ∼42 N/m [6]. Further, the absorption of G sheet is nearly constant and equal to 2.3%. However, it is hydrophobic, cannot be dispersed in polar solvents, has poor solubility, and is difficult to be processed in solution, which has hindered its applications.

G can be manufactured by numerous techniques, including exfoliation, epitaxial growth, chemical vapor deposition (CVD), and chemical and electrochemical methods [7]. Mechanical exfoliation was the first method established to separate monolayer G by peeling it off from graphite flakes using a Scotch tape. This process yields G sheets of high quality although it is not appropriate for mass production.

Epitaxial growth is a substrate-based technique in which G is grown on a SiC single-crystal by thermal treatment under vacuum at high temperature (~1300 °C). The thickness of G layers can be tailored by controlling temperature and time, and the uniformity of the thickness improves with an annealing process [8].

Chemical vapour deposition (CVD) processes are the most suitable, simplest, and cost-effective to manufacture G on a large scale. G is straightforwardly grown on a substrate made of a transition metal (Cu, Ni, Pt, Pd, Ru, Ir) via saturation of carbon upon exposure to a hydrocarbon gas (i.e., methane) at a high temperature [4]. As the substrate cools, the solubility of carbon on the substrate decreases and the carbon precipitates to form mono- to multilayer G sheets. The main drawbacks are the challenging control of the film thickness and the requirement of expensive substrates.

Chemical solution processes are low-cost large-scale production methods to synthesize G [8]. The main limitation of these methods is the insolubility of G. Nonetheless, the reaction with strong oxidizing agents, such as H_2_SO_4_, HNO_3_, or KMnO_4_, results in the anchoring of oxygen functional groups to both the basal plane and the edges of G sheets, leading to GO (Figure 1), which is soluble in numerous solvents, such as water, dimethylformamide (DMF), tetrahydrofurane (THF), and chloroform [9]. Due to its dispersibility in common organic solvents, GO is highly suitable as a filler in polymeric nanocomposites.

Another approach, scarcely used, is electrochemical exfoliation, based on the penetration of graphite by ions from the solution forced by the applied potential. G obtained by this method can be dispersed in organic solvents such as DMF, which enables the fabrication of thin films [8]. After reduction via treatment with HNO_3_ or under vacuum, layers with good transport and optical properties can be obtained.

GO is an oxidized form of G that contains epoxides, hydroxyls, and carbonyls on the basal planes and carboxyls on the edges (Figure 1). Accordingly, some properties of GO differ from those of G [9]: The sp^3^ carbon atoms in GO increase the interlayer spacing, improving its ability to retain compounds. The attached groups and lattice defects modify the electronic structure of G and serve as strong scattering centers that affect the electrical transport. Thus, it presents significantly lower electron mobility (ca. 0.1 cm^2^/V s), and it is typically insulating, with a sheet resistance of about 10^12^ Ω/sq or higher [10]. It has a low thermal conductivity of about 1 W/mK, and its mechanical properties depend on the number of defects and thermal treatment, with a Young modulus ranging from 200 GPa to 1 TPa [11]. Moreover, it shows aqueous processability, amphiphilicity, surface functionalization capability, and versatility. More importantly, it is highly hydrophilic and can form stable aqueous colloids to facilitate the assembly of macroscopic structures [12], which is crucial for large-scale uses.

GO can be manufactured by “bottom-up” and “top-down” methods. The first ones, in which simple carbon molecules are used, like those described above for pristine G, are time-consuming and face problems of scalability. Thus, top-down strategies are widely employed, including four main approaches [10]: Staudenmaier, Hofmann, Brodie, and Hummers. The most employed, the Hummers´ method, consists of the addition of KMnO_4_ to a solution of graphite, NaNO_3_, and H_2_SO_4_. Many variations of this method have been reported, with improvements constantly being explored to achieve better results and cheaper processes. For instance, approaches without using NaNO_3_ eliminate the evolution of NO_2_/N_2_O_4_ toxic gasses and simplify the disposal of waste water because of the inexistence of Na^+^ and NO_3_^−^ ions [13].

GO can also be synthesized from graphite oxide by using sonication, stirring, or a combination of both. Sonication is a time-effective way of fully exfoliating graphite oxide, although it can seriously harm the graphene flakes, reducing their size from microns to nanometres, and even producing graphene platelets. Mechanical stirring is a less heavy-handed approach, albeit it involves longer periods of time.

GO can be partly reduced to graphene-like sheets by eliminating oxygen-containing groups with the recovery of a conjugated structure (Figure 1). The rGO sheets are considered as functionalized G or chemically modified G [10]. The aim is to attain graphene-like materials comparable to the pristine G obtained from direct mechanical exfoliation of graphite both in structure and properties. However, residual functional groups and defects considerably modify the structure of the carbon plane; therefore, the properties of rGO differ from those of G. In particular, the electrical conductivity of rGO is typically in the range of 10–23 S/cm, and its thermal conductivity is about 60 W/m × K [14], much lower than that of pristine G.

The rGO synthesis can be performed by chemical, thermal, or photochemical methods. The thermal annealing consists of the reduction of GO by means of rapid heating (>2000 C/min) [15]. The exfoliation is caused by the evolution of CO or CO_2_ gases within the spaces between graphene sheets during the heating. This method cannot be used for GO films on substrates with a low melting-point, such as glass and polymers. Thermal annealing can also be accomplished by microwave irradiation or via photo-reduction [16], using the energy emitted by a flash lamp or a laser. This procedure can result in higher level of GO reduction, as the lamp/laser can offer higher energy than thermal annealing, leading to rGO films with higher conductivity [17].

Reduction by chemical reagents relies on their chemical reactions with GO, and can be performed at room temperature or on applying moderate heating. The reagent more typically used is hydrazine monohydrate [18]. However, this is a toxic and explosive chemical; hence, other “green” reducing agents have been proposed like ascorbic acid [19], sodium citrate [20], dopamine [21], sugar, aminoacids, etc. Electrochemical reduction is also possible, and does not require chemical reagents. The reduction is exclusively driven by the electron exchange between GO and the electrodes in a conventional electrochemical cell [22].

Regarding the biocompatibility of graphene-based materials, that is, their ability to interact with the living body, tissues, and cells without harmful effects, controversial results have been reported [23]. Their interactions with living cells depend on a large number of factors, including their hydrophilicity, purity, level of functionalization, lateral size, layer number, and so forth [24,25]. Thus, they can damage cell membranes, comprising phospholipids and cholesterol, resulting in potential cytotoxic effects. Pristine graphene cannot bind the phospholipids via electrostatic interactions, while can interact with their fatty acid chains by means of hydrophobic interactions [24]. In contrast, GO can electrostatically interact with the phospholipids since it possesses oxygenated functional groups on its surface. Besides, these nanoscale carbon materials can penetrate the cytoplasm, causing the leakage of cytoplasmic content, mitochondrial disorders, and lipid peroxidation. Moreover, if they enter the nucleus, they can subsequently react with DNA and induce genotoxic effects [26]. Most studies state that rGO is less toxic than GO [26], due to the reduction of functional groups. However, a few investigations have shown that rGO is more harmful to some cells like glioma ones [27]. Cytotoxicity can be minimized by attachment onto GO or rGO surfaces of biocompatible polymers incorporating amine and quaternary ammonium groups like chitosan (CS) [28], polyethyleneimine (PEI) [29], or poly(L-lysine) (PLL) [30]. Although the potential of GO for biomedical purposes has been clearly demonstrated, the lack of live cell compatibility information has limited its practical applications.

## 2. Antibacterial Action of Graphene and Its Derivatives

The defects contained in GO and rGO sheets strongly influence not only their physical properties but also their chemical and biochemical. Hence, the nanomaterial-bacteria interactions are conditioned by the type of graphene-based nanomaterial. Further, depending on the synthesis process, the number of oxygenated groups including hydroxyl, ketone, epoxy, ether, and carboxylic acid will vary, resulting in C/O ratios ranging from 0.43 [22] to 25.3 [31]. The degree of hydrophilicity plays a crucial role in antibacterial activity. G and rGO are hydrophobic, and their nanosheets tend to aggregate by means of π–π stacking interactions, favoring a strong and rapid adsorption of the bacteria on their surfaces. In contrast, GO is hydrophilic and amphiphilic, and bacterial adhesion has been reported to be slower, following a reversible model [32]. Besides, other parameters such as the nanomaterial surface charge, sheet size, and shape also condition the antibacterial activity. In this regard, G presents a neutral surface charge, while GO and rGO display negatively charged surfaces due to the deprotonation of the hydroxyl and carboxylic acid groups, and this influences bacterial adhesion. In addition, the sheet size can vary from a few nanometers to several micrometers, and the antimicrobial activity has been found to decrease as the size increases (ca. 4-fold increase in the antimicrobial activity when GO sheet area decreased from 0.65 to 0.01 μm^2^ [33]). It has also been demonstrated that insertion of G nanosheets within the lipid bilayer is size-dependent [34], and that microscale-sized graphenes tend to adopt a near-perpendicular configuration with respect to the cell wall, whereas nanosheets prefer a parallel position to the lipid bilayer. This is motivated by the preferential attraction between the hydrocarbon tails of the lipids and the lipophilic and flat G surface that allows the nanomaterial to sink in amongst the lipid tails, embedding the G nanosheets in the cell membrane. Further, it has been shown that the smaller the G layers are, the more freely they can diffuse into the lipid membrane in a preferential perpendicular orientation, whereas larger sheets prefer to organize themselves across the membrane, embedding themselves in the more lipophilic section of the cell membrane [35]. In addition, other G features like surface topography and roughness can also condition the antibacterial activity [36].

**Figure 2:**
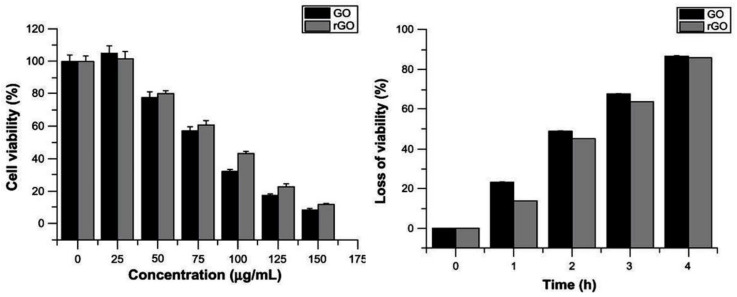
Antibacterial action of graphene oxide (GO) and reduced graphene oxide (rGO) in *Pseudomonas aeruginosa*. Reproduced from Ref. [44], with permission from Dove Medical Press, 2012.

Over the last decade, many works have focused on the antibacterial activity of GO and rGO dispersions [37,38,39,40,41,42,43,44]. The pioneer study by Liu and coworkers [37] provided information on the antibacterial activity of graphitic-based materials against *E. coli*, highlighting that GO displayed the highest action, followed by rGO, graphite, and graphite oxide. In most of the studies, as expected, the antibacterial action increased with increasing time and concentration (Figure 2), the bactericide effect being stronger against Gram-positive bacteria.

The lateral dimensions and level of nanomaterial aggregation also play a key role in the antibacterial activity of graphene-based materials. Well-dispersed sheets display stronger activity, since they can cover the cells more easily. Thus, it was found that bacteria can be wrapped by a thin layer of GO, which inhibits cell proliferation, resulting in significant cell viability loss [40]. In contrast, bacteria cells are typically embedded within large rGO aggregates that lead to lower efficacy. Conversely, conductive rGO and G show higher oxidative potential than insulating GO, which would result in a higher bactericide action [38].

**Figure 3:**
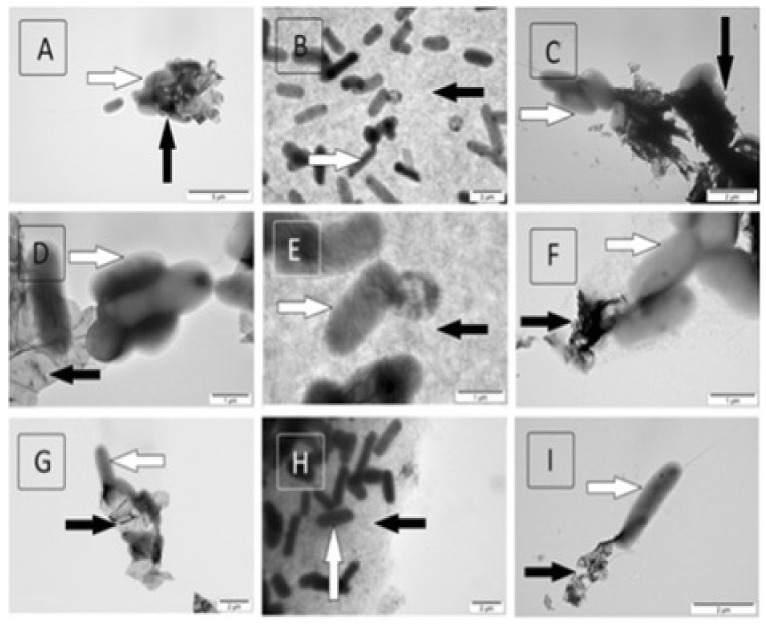
TEM images showing the interaction of graphene-based materials with *Salmonella enterica*. Pristine graphene (**A**, **D**, **G**), graphene oxide (**B**, **E**, **H**), and reduced graphene oxide (**C**, **F**, **I**). Reproduced from Ref. [41], with permission from SpringerOpen, 2015.

Different mechanisms have been reported to explain the antibacterial activity of graphenic materials, namely chemicals such as production of reactive oxygen species (ROS), oxidative stress at the bacterial membrane or extraction of a large amount of phospholipids from the cell membrane, as well as physically induced by the interaction of the sharp edges of graphene with bacterial membranes (resulting in a loss of cell membrane integrity), photo-thermal ablation, and mechanical wrapping, causing cell lysis [12,41,44]. The action could comprise of three stages (Figure 3): (i) cell deposition onto the graphene material, (ii) membrane stress induced by direct contact with sharp nanosheets, and (iii) superoxide anion-independent oxidation.

The lipid extraction mechanism has been confirmed by TEM by simulating the interaction between GO and both the outer and inner bacterial membranes (Figure 3). The strong van der Waals interactions between the GO nanosheets and the membrane lipids cause their extraction. Once extracted, hydrophobic interaction plays a leading role wherein the lipid hydrophobic tails spread out mainly on the unoxidized hydrophobic regions of GO, whereas the hydrophilic head groups prefer to contact polar oxide functions via electrostatic interactions. Therefore, both the GO insertion and lipid extraction induced severe membrane stress and reduced cell viability, a process that has been found to be concentration-dependent and increases with increasing GO lateral size.

The interaction of the edges of G nanosheets with bacterial membranes is frequently called the “insertion mode of action”. The G sharp edges can act as knives to cut through the bacteria’s cell membranes, leading to the leakage of intracellular substrates, thereby causing cell death [38]. Both GO and rGO also display bactericidal behavior towards Gram-negative and Gram-positive bacteria via this mechanism.

**Figure 4:**
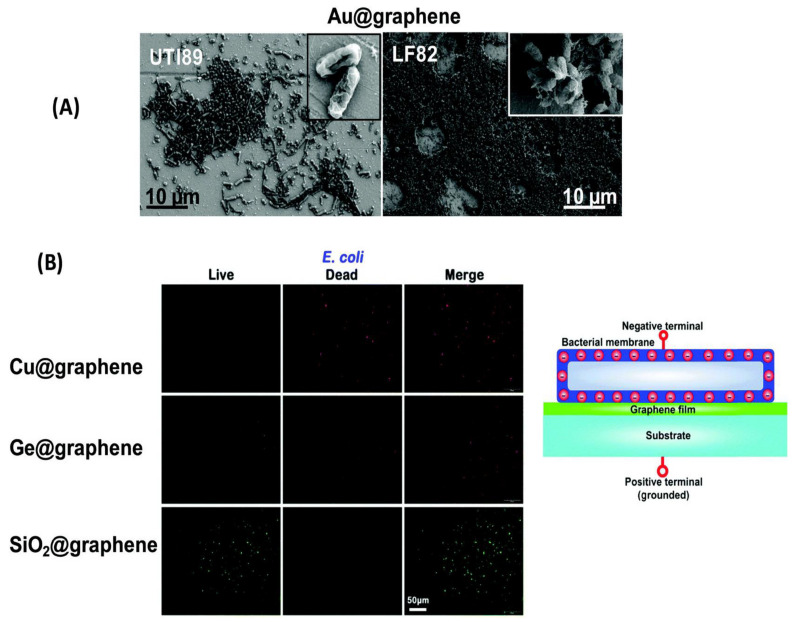
(**A**) SEM micrographs of *E. coli* UTI89 and LF82 on gold coated with CVD graphene; (**B**) fluorescence images showing the viability of bacteria after 24 h incubation over different interfaces (live bacteria are green, dead ones are red) and a schematic representation of the proposed mechanism. Reproduced from Ref. [42], with permission from Nature Research Publishing, 2014.

Related to the insertion mode of action is another mechanism that stipulates that the destructive effect of G on the bacterial membrane is induced by “direct contact” with the basal plane of the nanomaterial [42,45]. In this regard, the adhesion behavior of two different *E. coli* strains, UTI89 and LF82, towards gold interfaces coated with CVD graphene has been investigated (Figure 4). As can be observed from the SEM micrographs (Figure 4a), neither morphological changes nor membrane damage of the bacteria took place, indicating that such interfaces do not have antibacterial properties. The antibacterial activity of monolayer graphene on Cu, Ge, and SiO_2_ has also been investigated [42], and it was found that films on Ge and especially on Cu strongly restricted the proliferation of both *S. aureus* and *E. coli*. However, graphene films on SiO_2_ could not inhibit their growth, demonstrating that this nanomaterial has bactericide action on conducting and semiconducting surfaces. Further work [45] demonstrated that the antibacterial efficiency of G-based nanomaterials is not dependent on its sharp edges but on the contact that occurs between the nanomaterial basal plane and the bacteria cells. Thus, masking the GO basal plane decreased its antimicrobial efficiency by decreasing the extent of direct contact with the bacteria [46].

Another potential mechanism of G antibacterial action could be that its hydrophobic sheets perturbed the protein–protein bonding in the cell membrane, inducing the destabilization of the 3D structure of the protein, hence inducing a self-killing effect [15].

## 3. Antibacterial Action of Nanoparticle–Graphene based Nanocomposites

While graphene-based materials exhibit antibacterial properties, they have a strong tendency to agglomerate, in particular G and rGO, due to strong van der Waals interactions among the sheets, which condition their antibacterial action. This issue can be prevented via formation of nanocomposites by surface modification with metal ions/oxides NPs. Inorganic NPs can enhance graphene properties due to their high surface area-to-volume ratio, which leads to novel chemical, electrical, mechanical, optical, and electro-optical properties that differ from those of their bulk counterparts. Further, the mixing of G and rGO with metal oxide NPs can improve their dispersibility in polar solvents such as water [10]. Thus, numerous nanocomposites have been developed over recent years that combine the bactericide action of inorganic nanoparticles with the inherent antibacterial property of graphene-based nanomaterials to attain synergistic effects, and the most representative examples will be reviewed in the following sections.

### 3.1. Nanocomposites with Silver Nanoparticles

The antibacterial properties of Ag^+^ and Ag-based composites have been comprehensively explored [47]. Ag^+^ can damage the bacterial membranes and also produce ROS via photocatalytic activation. Nonetheless, when Ag nanoparticles interact with bacteria, they agglomerate; hence their effective specific surface area decreases, leading to reduced antibacterial action [48]. To solve this issue, nanocomposites of graphene and Ag nanoparticles were developed [49,50], although the improvements in antibacterial activity were still limited. Therefore, numerous studies have been reported on GO and rGO nanocomposites comprising Ag nanoparticles [51,52,53,54,55,56,57,58,59,60,61,62] (Table 1), showing improved performance compared to GO and Ag nanoparticles alone due to synergistic effects; further, the mixture of both constituents considerably reduces the concentrations required to inhibit all bacteria. Thus, significantly higher antibacterial activity was found for AgNPs-GO composites (1:2 ratio), with an average NP size of 80 nm [52] at a concentration of 200 μg/mL (Table 1), compared to that of GO or AgNPs, attributed to a higher ROS production, hence resulting in stronger oxidative stress and disruption of the cell membrane. Further, the AgNPs attached to GO are more stable and well-dispersed, which also contributes to the higher efficacy. Comparable antibacterial action has been found for similar composites with smaller nanoparticles (ca. 40 nm) for an optimal AgNPs:GO ratio of 1:1 even at concentrations as low as 2.5 μg/mL [58].

**Figure 5:**
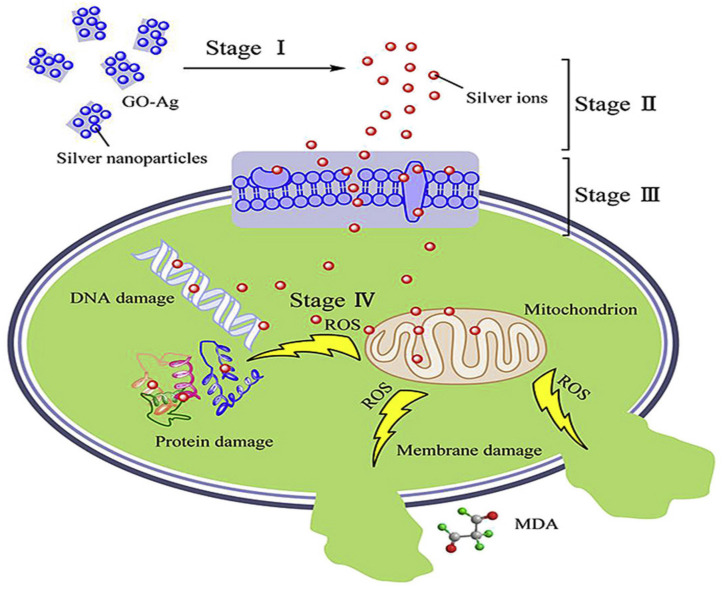
Representation of the antimicrobial action of AgNP-GO nanocomposites. Reproduced from Ref. [60], with permission from Elsevier, 2016.

Bacterial cell disruption seems to be the main mechanism against Gram-negative bacteria, while the inhibition of cell division could account for the lysis of Gram-positive ones. AgNPs-GO nanocomposites cause stronger damage towards *E. coli* membrane compared to *S. aureus*, as can be observed in Table 1. Thus, the antimicrobial potential of these nanocomposites is also influenced by the thickness of the cell wall of the microorganisms. The wall of Gram-positive contains a thick layer (20–80 nm) of peptidoglycan attached to teichoic acids. In Gram-negative bacteria, the cell wall comprises of a thin (7–8 nm) peptidoglycan layer and an outer membrane. The thicker peptidoglycan layer in Gram-positive bacteria could account for their higher resistance towards the effects of AgNPs-GO. Their bactericide action can be depicted in four stages (Figure 5) [60]: release of Ag^+^ ions, penetration through the cell membrane, ROS generation, followed by DNA, protein, mitochondrion, lipids and membrane damage, which finally leads to cell death.

Lipids constitute a main target during oxidative stress. Free radicals can directly attack the polyunsaturated fatty acids in the bacteria, yeast their membranes, and activate peroxidation of lipids, resulting in a drop in membrane fluidity, which can significantly disrupt membrane-bound proteins. DNA is also a key target. Mechanisms of DNA damage include abstractions and addition reactions by free radicals, leading to carbon-centered sugar radicals and OH- or H-adduct radicals of heterocyclic bases. The sugar moieties creating single- and double-strand breaks in the backbone, adducts of base and sugar groups, and cross-links to other molecules can block replication [63].

It is worthy to mention that AgNPs-rGO nanocomposites as antibacterial agents are more limited due to the tendency of the nanosheets to aggregate via strong van der Waals interactions. While the interaction between G (or rGO) and Ag^+^ should be mainly via hydrophobic and van der Waals forces, Ag^+^ nanoparticles have been reported to interact with the negatively charged oxygen-containing functional groups on the GO surface via electrostatic binding [55]. The AgNP-GO nanocomposites showed improved colloidal stability, photo-stability, and antibacterial activities against Gram-negative bacteria compared to AgNPs alone. GO plays a key role in improving the stability of the AgNPs, acting as a platform to prevent their agglomeration. Moreover, composites comprising smaller AgNPs showed better antibacterial activity than those with bigger NPs. This effect of the nanoparticle size has been further corroborated by different authors. Thus, the antibacterial efficacy of AgNP-GO with four NP sizes (10, 30, 50, and 80 nm) against *E. coli* and *S. aureus* has been tested, and its was found that the composite with the smallest NP showed the best activity [61]. Similarly, a recent study [62] investigated the bactericide action of AgNP-GO (ratio 1:2.3) manufactured via an environmentally friendly one-step approach, which comprised of spherical NPs with different diameters. The smallest NPs (ca. 3.1 nm) were obtained for the lowest concentrations of the silver precursor and the lowest synthesis temperatures, and led to an exceptional antibacterial action against Gram-negative *E. coli* and *P. aeruginosa*, as well as Gram-positive *S. aureus* and *C. albicans* (Table 1). Small-sized AgNPs have a larger surface area, resulting in more efficient cell–particle contact.

### 3.2. Nanocomposites with Gold Nanoparticles

The antibacterial activity of gold NPs also depends on their size and shape. Those of smaller dimensions (ca. less than 2 nm) are more likely to penetrate the bacterial cells and cause cell damage, followed by death [64]. Further, triangular-shaped NPs display better activity towards Gram-positive and Gram-negative bacteria than spherical ones [65], since their sharp edges can pierce the membranes of endosomes and translocate to the cytoplasm where they can be retained. The NPs can anchor to the bacterial membrane by electrostatic interaction and disrupt its integrity. They can alter the membrane potential and reduce the ATP levels within the cell, inhibiting the binding of tRNA with ribosomal subunit and thus disturbing translation [66]. Further, Au NPs can produce holes in the cell wall causing leakage of cell contents, and bind with the DNA, hindering transcription. They are also able to generate oxidative via free radical formation: the interaction between small NPs and bacteria probably induces a metabolic imbalance in the bacterial cell, resulting in an increase of ROS production that concluded in bacteria death [64].

**Figure 6:**
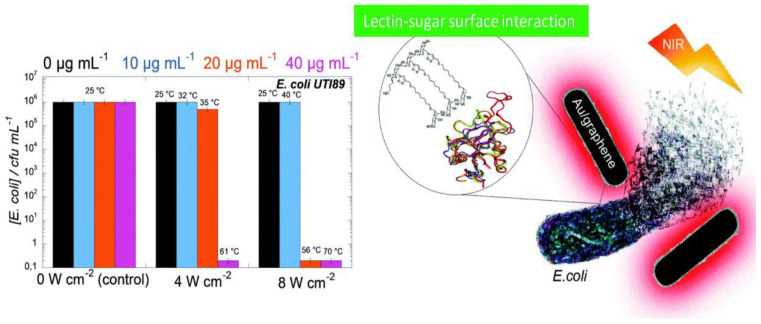
*E. coli* viability in the presence of rGO–PEG–Au NPs upon irradiation at 4 or 8 W cm^−2^ for 10 min. Reproduced from Ref. [68], with permission from the Royal Society of Chemistry, 2015.

Au nanoparticles have also been integrated with GO and rGO [67,68]. These composites also possess higher antibacterial activity than the individual components, and the bacterial cell disruption seems to be induced by the leak of sugars and proteins from the cell membrane when it comes in contact with the nanocomposite [67]. Further, Au nanostructures wrapped by rGO modified with polyethylene glycol (PEG) have been recently applied for the photothermal ablation of bacteria [68], and optimal inhibition of *E. coli* was attained at high temperatures (56–70 °C), Figure 6. This novel biocompatible process for pathogen ablation opens up a new perspective for treatment of urinary infections.

### 3.3. Nanocomposites with Cooper Oxide Nanoparticles

A few studies have also been devoted to investigating the antibacterial action of Cu [69], Cu_2_O [70,71] or CuO [72] nanoparticles bound to GO or rGO. At high concentrations, these nanomaterials are toxic to the majority of microorganisms since the redox properties of Cu provoke cellular damage including protein and lipid oxidation. Thus, it is crucial to control the release of Cu^2+^ ions, which produce ROS species on the bacterial surface. Cu_2_O can interact with GO and rGO nanosheets via physisorption, electrostatic attraction, or charge-transfer. The nanocomposites have improved bactericidal properties than each individual component, since the graphenic nanosheets can stimulate the Cu_2_O to generate more ROS [71], and also prevent nanosheet aggregation. Simultaneously, rGO prevents Cu_2_O from reacting with the environment and releasing Cu^2+^ rapidly.

**Figure 7:**
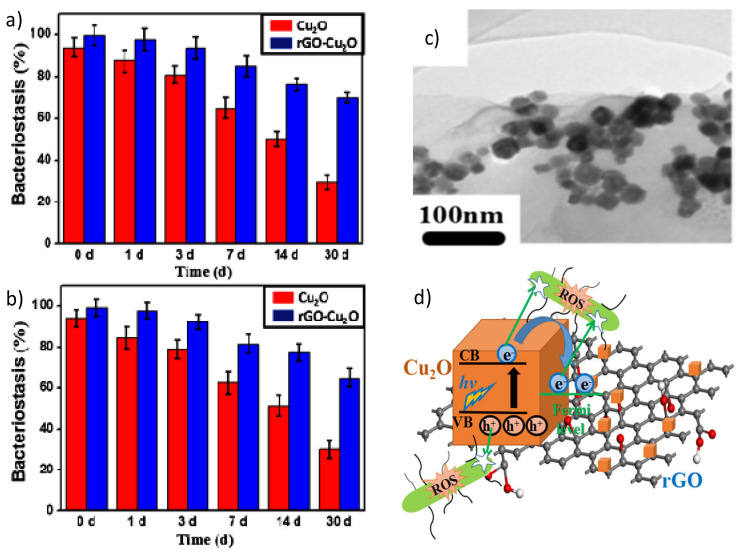
Percentage of bacterial reduction of *E. coli* (**a**) and *S. aureus* (**b**) in the presence of Cu_2_O nanoparticles and the Cu_2_O-rGO nanocomposite after immersion in phosphate buffered saline (PBS) for different days. TEM image of the Cu_2_O-rGO nanocomposite (**c**). Schematic representation of the mechanism of ROS production of the Cu_2_O-rGO nanocomposite (**d**). Reproduced from Ref. [71], with permission from Elsevier, 2019.

In a recent study [71], the antibacterial properties of Cu_2_O nanoparticles and a rGO-Cu_2_O nanocomposite were compared as a function of time (Figure 7a,b). When the samples were stored in phosphate buffered saline (PBS) for less than seven days, the nanocomposite showed about 14% higher antibacterial activity against *E. coli* and *S. aureus* than the Cu_2_O nanoparticles, which only produced ROS until the third day, while the nanocomposite systematically produced ROS during this period. For longer periods, the activity of the nanocomposite was considerably higher than that of Cu_2_O (i.e., 40% and 35% better against *E. coli* and *S. Aureus* after one month). The antibacterial mechanism seems to be based on ROS generation (Figure 7d). Upon light illumination, the photoinduced electron would be transferred from the Cu_2_O to the rGO, which could suppress the recombination of electron-hole pairs. The rGO could accept the photoexcited electrons from Cu_2_O, enabling improved charge transfer between the bacteria and the rGO-Cu_2_O nanocomposite [70].

**Figure 8:**
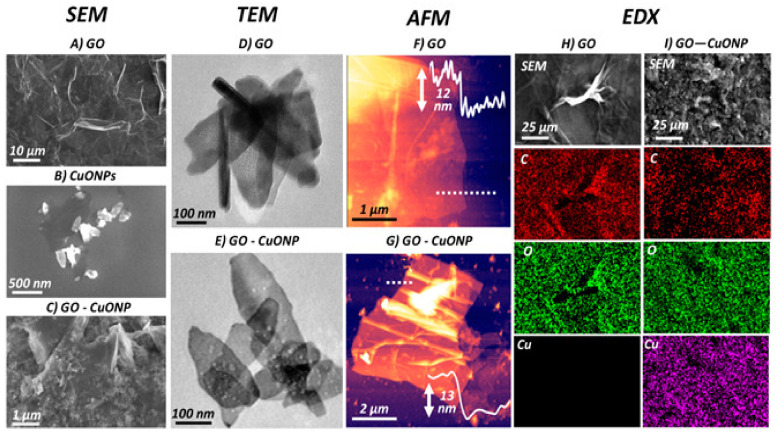
SEM images of (**A**) GO, (**B**) CuO nanoparticles, and (**C**) GO-CuO nanocomposite. (**D** and **E**) TEM and (**F** and **G**) AFM images of GO and GO-CuO, respectively. Energy-dispersive X-ray (EDX) spectral map for H) GO and I) GO-CuO nanocomposite. Reprinted from Ref. [72], with permission from the American Chemical Society, 2019.

Nanocomposites of CuO nanoparticles and GO have also been developed [72], which were found to be effective antibacterial nanomaterials, strongly inhibiting the proliferation of both *E. coli* and *S. typhimurium* bacteria compared to GO or CuO alone. The surface morphology of the nanocomposite significantly differed from that of the pure components (Figure 8), as revealed by scanning electron microscopy (SEM), transmission electron microscopy (TEM), and atomic force microscopy (AFM), and this could account for the improved bactericide action. An inspection of the nanomaterial surface (Figure 8c,e) shows the underlying sheets of GO covered by a high concentration of well-dispersed ellipsoidal NPs, with the short and long axis dimensions being ~80 nm and ~190 nm, respectively. AFM imaging (Figure 8f,g) revealed that the GO and GO-CuO flakes were about 12 and 13 nm in thickness, respectively, indicating that the nanocomposite is composed of a few layers of GO covered by a thin CuO coating. The energy-dispersive X-ray (EDX) mapping confirmed that the material on the modified GO contains cooper and oxygen.

### 3.4. Nanocomposites with Titanium Oxide Nanoparticles 

TiO_2_ is an important semiconducting material due to its chemical inertness, non-toxicity, low cost, excellent chemical/thermal stability, high UV absorption, and strong antibacterial activity against a large variety of microorganisms [73,74,75]. A few works have been recently reported on the photocatalytic antibacterial properties of GO and rGO nanocomposites incorporating TiO_2_ [76,77,78,79], which provide a green and effective method for the inactivation of various microorganisms by generating ROS. The photocatalytic activity of TiO_2_ improves after coupling with G derivatives, attributed to the remarkable electron conductivity of the carbon nanomaterial that provides a 2D network reservoir to accept as well as shuttle photogenerated electrons from the semiconductor, which results in the separation and prolonged lifetime of hole-electron pairs [80]. Further, GO can interact with TiO_2_ via H-bonding and polar forces, while in the case of rGO, the hydrophobic interactions play a dominant role. The antibacterial activity of a GO-TiO_2_ nanocomposites against E. coli. was investigated, and a complete inactivation was found under light irradiation for 30 min at a concentration of 180 μg/mL [77], Table 1. The inexpensiveness of TiO_2_ and the easiness of manufacturing magnetic GO–TiO_2_ make them suitable for water disinfection treatment. In addition, cotton fabrics have been covered with rGO decorated with TiO_2_ nanoparticles in order to provide self-cleaning characteristics and improve the antimicrobial properties [78], and the nanocomposite coating strongly restricted the growth of E. faecalis and S. aureus. In general, studies indicate higher activity of rGO-TiO_2_ against Gram-positive bacteria compared to Gram-negative ones. rGO-TiO_2_ nanocomposites have also been developed for the synergetic degradation of fluoroquinolone in a pulse discharge plasma (PDP) system [79], and the highest removal efficiency (99.4%) was attained at 5 wt% rGO content, which was 23.7% higher than that for TiO_2_ alone.

### 3.5. Nanocomposites with Zinc Oxide Nanoparticles

ZnO is another semiconductor photocatalyst widely used as an antibacterial agent [81,82]. Its properties depend mainly on its specific surface area and the number of surface sites at which reactions take place with absorbed molecules. The release of Zn^2+^ has been proposed as one of the principal antibacterial mechanisms of these nanoparticles, together with the penetration and disruption of the bacterial membrane [83]. Nonetheless, as occurs with other nanoparticles, agglomeration hinders efficient antibacterial action. The mixing of GO or rGO with ZnO nanoparticles can prevent their aggregation, leading to nanocomposites with good environmental stability and better antibacterial properties than each of the components alone [84,85,86,87,88,89,90,91]. ZnO-GO nanoparticles have been prepared via a simple one pot reaction [87] in which the GO aided the ZnO dispersion, slowed down the ZnO dissolution and acted as an anchorage point, favoring the direct intimate contact bacteria-ZnO.

**Figure 9:**
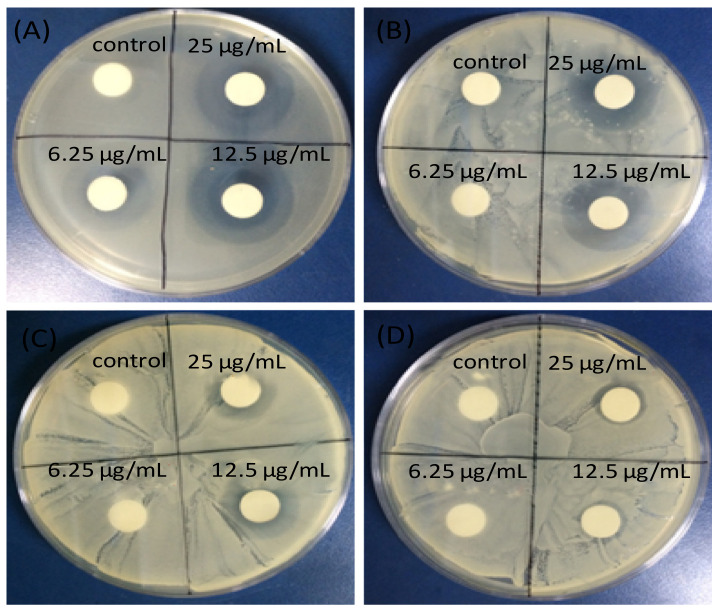
Inhibitory zones of ZnO-GO nanocomposite against (**A**) *Escherichia coli*; (**B**) *Salmonella typhimurium*; (**C**) *Bacillus subtilis*; (**D**) *Enterococcus faecalis*. Adapted from Ref. [91], with permission from Dove Medical Press, 2015.

The mechanism of photocatalytic inactivation of E. coli by GO-ZnO nanocomposites has also been investigated [88], and it was reported that GO promoted the charge transfer, favoring the bulk production of ROS, hence improving the bacterial inactivation efficiency. ZnO/graphene quantum dot nanocomposites have been synthesized via the hydrothermal method [90] and showed enhanced antibacterial activity on E. coli compared to the individual components, attributed to superior ROS production under UV-irradiation. Further, ZnO nanoparticles on GO [91] were able to inhibit the growth of *E. coli*, *S. typhimurium*, *B. subtilis,* and *E. faecalis* bacteria (Figure 9), after 24 h of incubation. Excellent antibacterial activity of the nanocomposite can be observed with minimum inhibitory concentrations (MIC) of 6.25 μg/mL for *E. coli* and *S. typhimurium*, 12.5 μg/mL for *B. subtilis*, and 25 μg/mL for *E. faecalis*, significantly lower than those found for the individual components. This proves that GO-ZnO nanocomposites at low concentration can be effectively used to inhibit the growth of bacteria.

This enhanced behaviour is ascribed to cell membrane disruption due to the release of the Zn^2+^ ions and the ROS production. Thus, Zn^2+^ can link to the negatively charged bacterial membrane, causing the protein on the membrane to solidify, thus inhibiting the bacterial proliferation. Besides, electrons can be quickly transferred between the ZnO nanoparticles and the GO, and subsequently absorb surface oxygen to form ROS, resulting in the formation of lipid peroxide, consequently damaging the bacterial membrane.

The nanocomposite synthesis method also influences the antibacterial activity. Those prepared via electrophoretic deposition (EPD) showed better properties than those manufactured via drop-casting [84]. Thus, the EPD method led to effective penetration of the negatively charged GO sheets into ZnO nanowires to form a spider net-like structure, whereas the drop-casting technique caused only the surface coverage of the GO sheets onto the nanowires. The ZnO nanowires alone could just inactivate 58% of *E. coli*, while both drop-casting and EPD-prepared GO/ZnO nanocomposites displayed significant antibacterial activity; in particular, the EPD one caused 99.5% photoinactivation under visible light irradiation for 1 h at a concentration of 500 μg/mL, Table 1.

### 3.6. Multicomponent Nanocomposites

**Figure 10:**
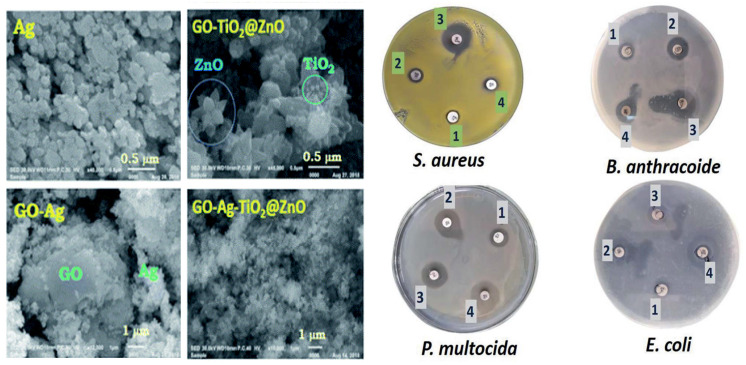
**Left**: SEM images of Ag, GO–Ag, GO–TiO2@ZnO, and GO–Ag–TiO2@ZnO nanocomposite. **Right**: Inhibitory zones of GO–Ag–TiO2@ZnO nanocomposite against *S. aureus, B. anthracoides* and Gram-negative *E. coli* and *P. multocida*. Adapted from Ref. [98], with permission from the Royal Society of Chemistry, 2019.

Several graphene-based multicomponent composites have been prepared by incorporating different nanoparticles with graphene or its derivatives, in order to attain improved antibacterial activity due to synergistic effects. For instance, GO-Ag-TiO_2_ nanocomposite coatings were synthesized using a hydrothermal process for the control of the food-borne pathogen *Campylobacter jejuni*. The nanocomposites efficiently inhibited the aggregation and growth of *C. jejuni* as well as biofilm formation [92]. Different nanocomposites incorporating iron oxide nanoparticles have also been developed [93,94,95,96,97], Table 1. Magnetic GO-MnFe_2_O_4_ hybrids at a concentration of 100 μg/mL led to about 82% inhibition on *E. coli* after only 2 h of contact [95]. Hybrids with polyethylenimine wrapped Ag nanoparticles and Fe_2_O_3_ caused 100% inhibition of *E. coli* at a concentration of only 0.1 μg/mL [93]. A multicomposite of GO, CoFe_2_O_4_, and Ag nanoparticles was also manufactured to disinfect water contaminated with *E. coli* and *S. aureus*, leading to almost complete inhibition at 12 μg/mL [96]. GO decorated with Ag, TiO_2_, and ZnO nanostructures has been prepared via ultrasonication and casting, and the antibacterial activity of the nanocomposite was tested against Gram-positive *S. aureus* and *B. anthracoides* and Gram-negative *E. coli* and *P. multocida* [98]. Figure 10 shows SEM images of GO, Ag, GO-Ag, GO-TiO_2_-ZnO, and GO-Ag-TiO_2_-ZnO nanocomposites.

As can be observed, the GO nanosheet was densely packed by the metal oxides, representative of a good combination between the carbon nanomaterial and the inorganic NPs. Thus, the GO nanosheets seem to act as bridges for the metal oxide NPs. Ag NPs have a spherical morphology and are located on the GO surface. The ZnO nanoflower appears over the GO surface, while the spherical TiO_2_ is deposited onto the ZnO nanoflower. The Ag NPs alone had the lowest inhibitory effect on all tested bacteria. The GO–TiO_2_-ZnO nanocomposite showed highly suppressing microbial growth against both Gram-positive and Gram-negative bacteria, while the four-component nanocomposite was found to be the most effective against Gram-negative bacteria (Figure 10). The improved activity of the hybrid was ascribed to the synergistic effect of the different components: direct damage of the cellular membrane by the Ag nanoparticles, ROS generation by the TiO_2_ and ZnO, contact between the GO sharp edges and the bacteria, and the accumulation of the nanoparticles in the cytoplasm.

Ag/ZnO/rGO nanocomposites with different weight ratios were synthesized via rapid microwave irradiation [99]. Depending on the composition, the hybrids were more effective against Gram positive or Gram negative bacteria, and the optimal performance was found at 7 wt% Ag and 15 wt% ZnO. Further, multifunctional nanocomposites of GO incorporating both Ag and Fe_2_O_3_ nanoparticles were prepared and characterized [100]. The MIC values against *E. coli* and *S. aureus* were 0.025 and 0.05 mg/mL, respectively These composites display both antibacterial and magnetic properties, and can be harvested with a magnet.

On the other hand, hydroxyapatite (HA) (Ca_10_(PO_4_)_6_(OH)_2_), has excellent biocompatibility, osteoconductivity, is non-toxic, and is widely used for biomedical applications [101]. rGO/HA/Ag multicomponent nanocomposites have been prepared using the hydrothermal method [102] and characterized via different spectroscopic techniques. They showed improved antibacterial activity against *B. cereus, S. aureus, E. coli* and *K. pneumonia* than the pure components.

Overall, the comparison of the data in Table 1 reveals that the optimum bactericide efficacy at the lowest concentration (0.1 μg/mL) is attained for the hybrid nanocomposite comprising Ag^+^ NPs, rGO and Fe_3_O_4_, with an antibacterial performance superior to those ever reported for photothermal materials, arising from the synergistic effect of the three components [93].

## 4. Concluding Remarks and Outlook

Selected examples about the progress on graphene-based antibacterial nanocomposites incorporating inorganic nanoparticles have been provided. It has been demonstrated that the nanoparticles present higher antibacterial activity compared with their bulk counterparts due to their higher surface-to-volume ratio, resulting in improved contact with microorganisms. The antimicrobial properties of the nanocomposites have been usually tested versus E. Coli and S. Aureus as model microorganism. In this regard, ongoing research should focus on other bacteria such as S. Epidermidis, S. enteritidis, S. typhimurium P. aeruginosa, E. faecalis, B. subtilis, etc., in order to provide a better perspective on the general antibacterial ability of graphene-based materials. This would account for the increasing antibiotic resistance among various bacteria and their association as a severe hazard to worldwide public health.

Generally, these nanomaterials cause stronger damage towards E. coli compared to S. Aureus, which could be related to the structural and chemical compositional differences of the cell membranes. Gram-positive bacteria possess one cytoplasmic membrane and a dense wall comprising multilayers of peptidoglycan, whereas Gram-negative have a multipart cell wall structure, with a peptidoglycan layer between the outer membrane and the cytoplasmic membrane.

Although the disparity in nanomaterial concentrations, syntheses processes, antibacterial measurement methods, and so forth make the comparison of the antimicrobial effectiveness difficult, it seems that nanocomposites loaded with Ag nanoparticles are the most effective, followed by those incorporating photocatalytic nanoparticles like TiO_2_ and ZnO. Furthermore, various multicomponent materials have been developed by combining different NPs or metal oxide NPs, which lead to higher antibacterial activity than the individual components and at much lower concentration due to a synergetic effect.

Another issue related to these nanomaterials is the large number of parameters influencing the antibacterial properties, including size, shape, orientation, defects as well as surface functional groups. Well-dispersed nanomaterials show stronger antibacterial activity than the aggregated ones. Further, composites with small-sized NPs are more effective since they have larger surface area in contact with the bacteria. On the other hand, triangular-shaped NPs display better activity towards Gram-positive and Gram-negative bacteria than spherical ones, since their sharp edges can penetrate cell membranes more easily.

The antibacterial activity of these nanocomposites seems to be a combination of different mechanisms, including release of inorganic NP ions, penetration through the cell membrane, ROS generation, DNA, protein, mitochondrion, and lipid damage as well as bacteria cell disruption. The last mechanism appears to be the key mechanism against Gram-negative bacteria, while the DNA, protein, mitochondrion, and lipid damage, resulting in inhibition of cell division could account for the lysis of Gram-positive ones.

Nonetheless, although the antimicrobial mechanism of graphene-based nanomaterials has been the aim of a number of recent investigations, the field is still in its infancy, and much controversial data has been reported; hence, a deeper and comprehensive knowledge of the molecular mechanisms involved is required. The main challenge is obtaining reliable information on the interaction between bacteria and graphene-based nanomaterials, as well as the effect of different parameters including basal planes, lateral size, oxygen content, etc. Another challenge is to analyze the toxicity associated with them. Despite numerous studies on this topic, there are inconsistencies in the results and lack of universal acceptance criteria for the toxicity of graphene-based materials, which urgently have to be solved prior to their use in practical applications.

Notwithstanding the challenges, it is expected that the progress made by scientists in developing graphene-based nanocomposites as antibacterial agents will be productive in the future. We should all collaborate with novel ideas to address the associated challenges.

## Figures and Tables

**Table 1:** Antibacterial properties of nanocomposites with graphene-based materials and inorganic NPs (S: spherical; QS: quasi-spherical; E: ellipsoidal; C: cubic; T: triangular; NW: nanowire; ST: star).

Graphene Material	NP shape/ size (Φ, nm)	Bacteria Model	Concentration (μg/mL)	Inhibition (%)	Ref.
AgNPs-GO	S/93	*S. enteritidis*	200	61	[51]
AgNPs-GO	S/80	*E. coli*	200	89	[52]
AgNPs-GO	S/80	*S. epidermidis*	200	76	[52]
AgNPs-GO	S/80	*S. aureus*	200	81	[52]
AgNPs-GO	S/80	*C. albicans*	200	78	[52]
AgNPs-GO	S, QS	*E. coli/S. aureus*	100	100	[53]
AgNPs-GO	QS/40	*E. coli*	6.4	100	[55]
AgNPs-GO	QS/60	*E. coli/S. aureus*	10	100	[56]
AgNPs-GO	E/98	*E. coli/S. aureus*	45	100	[57]
AgNPs-GO	S/40	*E. coli*	2.5	80	[58]
AgNPs-GO	S/40	*S. aureus*	2.5	78	[58]
AgNPs-GO	S/3.1	*S. aureus*	64	100	[62]
AgNPs-GO	S/3.1	*C. albicans*	64	100	[62]
AgNPs-GO	S/3.1	*E. coli/P. aeruginosa*	128	100	[62]
AgNPs-rGO	QS/57	*E. coli*	40	100	[54]
AgNPs-rGO	S/12	*E. coli*	20	100	[59]
Au-rGO	T, S/50	*S. aureus/B. subtilis*	250	94	[67]
Au-rGO	T, S/50	*E. coli/P. aeruginosa*	250	50	[67]
Au-rGO	T, S	*E. coli*	10	99	[68]
Cu_2_ONPs-rGO	C/30	*E. coli*	40	70	[71]
Cu_2_ONPs-rGO	C/30	*S. aureus*	40	65	[71]
CuONPs-GO	E/80	*E. coli*	3.0	90	[72]
CuONPs-GO	E/80	*S. typhimurium*	3.0	99	[72]
TiO_2_-GO	S/30	*E. coli*	180	100	[77]
ZnO-GO	NW/75	*E. coli*	500	100	[84]
ZnO-GO	ST/150	*E. coli/P. aeruginosa*	6.5	100	[86]
ZnO-GO	ST/150	*S. aureus*	11.5	100	[86]
ZnO-GO	ST/150	*B. subtilis*	15.0	100	[86]
ZnO-G	S/20	*E. coli*	3.0	100	[89]
Ag-Fe_2_O_3-_rGO	QS	*E. coli*	0.1	100	[93]
Fe_2_O_3-_GO	S/225	*E. coli*	100	97	[94]
MnFe_2_O_4_-GO	S/170	*E. coli*	100	82	[95]
Ag-CoFe_2_O_4_-rGO	QS/75	*E. coli/S. aureus*	12	98	[96]
Fe_3_O_4_-GO	S/66	*E. coli*	300	91	[97]
